# An Algorithm for Enhancing the Image Contrast of Electron Tomography

**DOI:** 10.1038/s41598-018-34652-9

**Published:** 2018-11-12

**Authors:** Hao Wu, Xiaobo Zhai, Dongsheng Lei, Jianfang Liu, Yadong Yu, Rongfang Bie, Gang Ren

**Affiliations:** 1 College of Information Science and Technology, Beijing Normal University, Beijing, China; 2 The Molecular Foundry, Lawrence Berkeley National Laboratory, Berkeley, CA 94720 USA

## Abstract

Three-dimensional (3D) reconstruction of a single protein molecule is essential for understanding the relationship between the structural dynamics and functions of the protein. Electron tomography (ET) provides a tool for imaging an individual particle of protein from a series of tilted angles. Individual-particle electron tomography (IPET) provides an approach for reconstructing a 3D density map from a single targeted protein particle (without averaging from different particles of this type of protein), in which the target particle was imaged from a series of tilting angles. However, owing to radiation damage limitations, low-dose images (high noise, and low image contrast) are often challenging to be aligned for 3D reconstruction at intermediate resolution (1–3 nm). Here, we propose a computational method to enhance the image contrast, without increasing any experimental dose, for IPET 3D reconstruction. Using an edge-preserving smoothing-based multi-scale image decomposition algorithm, this method can detect the object against a high-noise background and enhance the object image contrast without increasing the noise level or significantly decreasing the image resolution. The method was validated by using both negative staining (NS) ET and cryo-ET images. The successful 3D reconstruction of a small molecule (<100 kDa) indicated that this method can be used as a supporting tool to current ET 3D reconstruction methods for studying protein dynamics via structure determination from each individual particle of the same type of protein.

## Introduction

In solution, protein particles travel in following the Brownian motion and their structures are vibrated in following the thermodynamics. Studying the dynamics of a type of protein requires a capability for 3D structure determination of an individual particle of this type of protein. Conventional structure determination approaches, including X-ray crystallography and cryo-electron microscopy (cryo-EM) single-particle 3D reconstruction, require an averaging process on thousands to millions of different particles of the same type of protein^1^. Averaging the structures from different particles without prior knowledge of their structure identity, or ignored the nature flexibility or fluctuation of the particles may lead to artifacts in protein structure determination such as blurring or eliminating the flexible domains. The typical flexble proteins include the double strand DNA (dsDNA)^2–5^, antibody^6–8^, lipoprotein and neuron proteins^9–14^. As an example of the IgG1 antibody, by enforced averaging the particle images of IgG antibody in single-particle 3D reconstruction^6–8^, one or two domains could disappeared.

An ideal approach to reveal the protein dynamics is to tracking the 3D structure changes on each individual particle of protein in liquid solution. Since no technique is available for imaging the 3D structure of an individual particle of protein in solution, the molecular dynamic (MD) simulation was often used to reveal the dynamics of an individual particle of protein in solution. An alternative approach to reveal the protein 3D structural dynamics is to determine the 3D structures of hundreds of individual particles that were frozen or fixed in a same time. The statistical analysis of the variety of the 3D structures from different particles can reflect the 3D dynamics of this type of protein.

Electron tomography (ET) is a powerful tool for imaging a targeted particle from a series of tilted viewing angles. The computerized image algorithms enable us to align the images and reconstruct them into a 3D density map. The 3D reconstruction resolution significantly depends on the accuracy of the alignment of the tilted images and the noise levels of the images. High noise level can decrease the accuracy of the image alignment. Unfortunately, in the experiment, the capability to reduce the noise level of the images was limited by the radiation damage and the dose tolerance limitation.

To decrease the noise level (increase the image contrast) without increasing the imaging dose or radiation damage, a computational approach was here introduced. The approach included edge-preserving smoothing filters proposed by L.P. Yaroslavsky in 1985^15^, as an image-optimizing tool widely used in computer vision. By smoothing images while effectively preserving edges, this method can enhance image contrast by decomposing an image into piecewise smooth layers and detailed layers^15^. In 1990, Perona and Malik adopted a diffusion process to realize semantically meaningful edges^16^. To further improve edge-smoothing capabilities, two non-linear Gaussian-filter-based edge-preserving methods were introduced by Aurich and Weule in 1995^17^ and by Smith and Brady in 1997^18^. Since then, more image decomposition methods have been reported based on those edge-preserving smoothing models^19^. In most cases, a combination of multi-scale operations was used^20^. The limitations of these methods include some halo artifacts existing near the boundaries from using previous models^21^. To overcome these limitations, several optimized edge-preserving smoothing filters have been reported^16,22–24^. Among these methods, the bilateral filter (BLF)^23^ and weighted least squares (WLS) filter^22^ are two of the most effective tools for multi-scale decomposition, especially for detail extraction and noise removal. Compared with the BLF filter, the WLS filter is more convenient, flexible and appropriate for extracting multi-scale details.

Here, we introduce a method to enhance the image contrast of ET by using this WLS filter as an edge-preserving smoothing filter. The model was more effective than the traditional edge-preserving decomposition-based detail manipulation method^22^. To validate the capability, we tested this method to enhance the low-contrast simulated images, real experimental cryo-EM and cryo-ET images, and real experimental negative staining (NS) ET images, as well as the real experimental cryo-positive staining (PS) ET images. The final 3D reconstructions showed that this method can be useful for the 3D structure determination of an individual particle of protein by ET.

## Overview of the Algorithm

**Figure 1:**
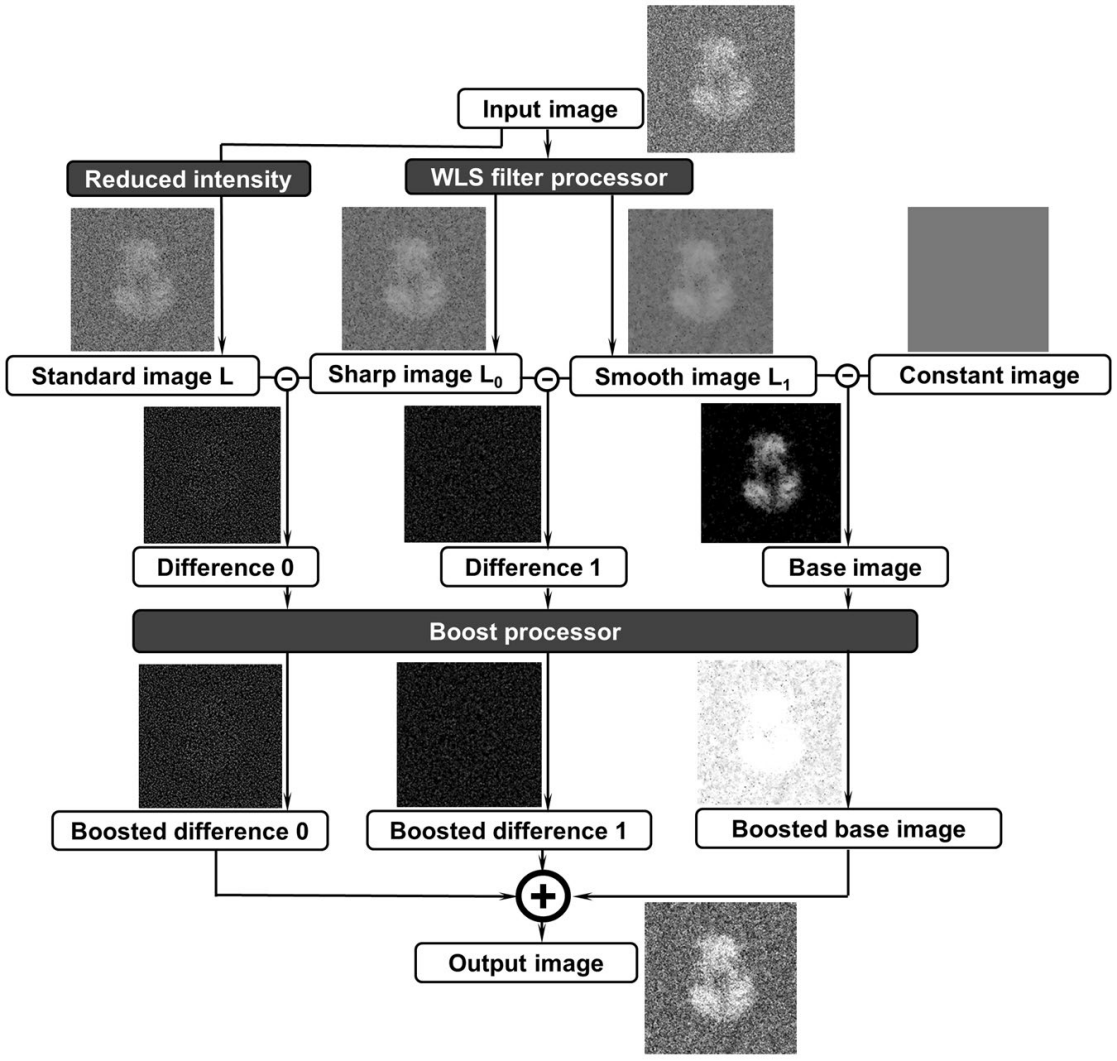
The flowchart and intermediate images of the image-contrast enhancement method using the edge-preserving smoothing-based multi-scale image decomposition algorithm. Image L is an optimized image after reducing the intensity for three times on the input image. Images L_0_ and L_1_ are two processed images filtered by sharp-scale and common-scale WLS filters, respectively. Constant image is a background image with the value of a magic number of 56. In order to show the details, above four images’ brightness and contrast were adjusted to 40 and 40 by Windows PowerPoint. The computed images, Difference 0, Different 1, Base images and their boosted images were adjusted to 80 in their image brightnesses, but without changing their image contrasts.

Flow chart of the algorithm of the edge-preserving smoothing-based multi-scale image decomposition for image contrast enhancement showed in Fig. 1 contains two processes, the WLS filter and image boosting. In the process of WLS filter, the input image was decomposed into 3 different images, standard image L, relatively sharp image L_0_ and relatively smooth image L_1_. L is an optimized image after reduced the intensity for 3 times on the input image. L_0_ and L_1_ are two processed images filtered by sharp-scale and common-scale WLS filters, respectively. Constant image was a blank image with a magic intensity of 56, which reflects the image’s background information. By subtracting from each other, three difference images, difference 0, difference 1 and base image were submitted for a boosting process for extracting the original image detail and base information at different dimensions. By combining those three boosted images into one, the output image can capture the major details in the different levels while maintaining the image’s base information. In the process, the WLS filter processer and boost processor as essential models can directly contribute to enhancing the image contrast, as presented in details in the following sections.

### The process of the WLS filter

Compared with other edge-preserving smoothing filters, the WLS filter^22^ has obvious advantages, especially for multi-scale decomposition and detail extraction. Through a WLS filter with appropriate parameters, a coarse, piecewise smooth version of decomposition can be obtained. Moreover, a sequence of images capturing different details at progressively finer scales is presented. As shown in Fig. 1, image *L*_0_ is obtained by using a sharp-scale WLS filter, and image *L*_1_ is obtained by using a common-scale WLS filter. Image *L*_0_ retains more details than image *L*_1_ (significant gradients). The constant image presents the background of the image with a constant intensity of 56 as a magic number used for any images. The differences between images *L*, *L*_0_ and *L*_1_ reflect the image details at different levels.

### The process of image boosting

As discussed above, the difference images reflect the image details at different levels (Fig. 1). However, we found that by directly calculating the differences, it was difficult to control the degree of detail in the layers. In some cases, the detailed layers were either significantly boosted or not obvious. Thus, this strategy to control the levels of detailed layers was challenging. In this case, we proposed an effective boosting function^25,26^ as follows:

1 $$y=\frac{1}{1+{e}^{-ax}}-0.5$$

where *x* indicates one input processed image, and *a* indicates the sigmoid parameter that determines the degrees of sigmoid. In our case, *a* = 8 was used. The boosting function above can be considered a sigmoid curve that can shift and normalize the target term. It not only contributed to controlling the contrast and exposure of the base layer but also contributed to keeping the boosting of details under controlling. A rescale process can be added later if necessary for the experimental data. The intermediate states of images of an example were used to show the changes of images before and after boosted process (Fig. 1).

We next applied the algorithm to both simulated and experimental images to test how the edge-preserving smoothing-based image decomposition models can effectively enhance the image contrast, in which some images were further evaluated by the 3D reconstruction.

### Implementation on the low-contrast 2D images

The enhancement algorithm was first tested on simulated 2D low-dose cryo-EM images. Considering the smaller protein is more challenging for imaging and 3D reconstruction, two proteins with different molecule weight were used: (i) a fragment (A-D chains, molecular mass: ~108 kDa) of a molybdate transporter (ModB_2_C_2_, PDB accession number 2ONK^27^) and (ii) cholesteryl ester transfer protein (CETP, PDB accession number 2OBD^28^, molecular mass: ~53 kDa). The simulated images of these two proteins were prepared by using the following protocol: (a) The simulated 3D density maps were generated by using the “*pdb2mrc*” command of the EMAN software package^29^, in which the map of the ModB_2_C_2_ protein was generated at a resolution of 4 Å within a box of 160 × 160 × 160 voxels (voxel size of 1 Å), and the map of CETP was generated at a resolution of 2 Å within a box of 192 × 192 × 192 voxels (voxel size of 1 Å). (b) The projection images of the maps were generated by using the “*PJ 3Q*” command of the SPIDER software package^30^. In the projections, each pixel was 1 Å. (c) To simulate noise in the cryo-EM image for evaluating the effect of enhancement method on images with different noise levels, Gaussian-type noises were applied to the above projections to achieve a final SNR of 0.80, 0.50 and 0.30 respectively.

**Figure 2:**
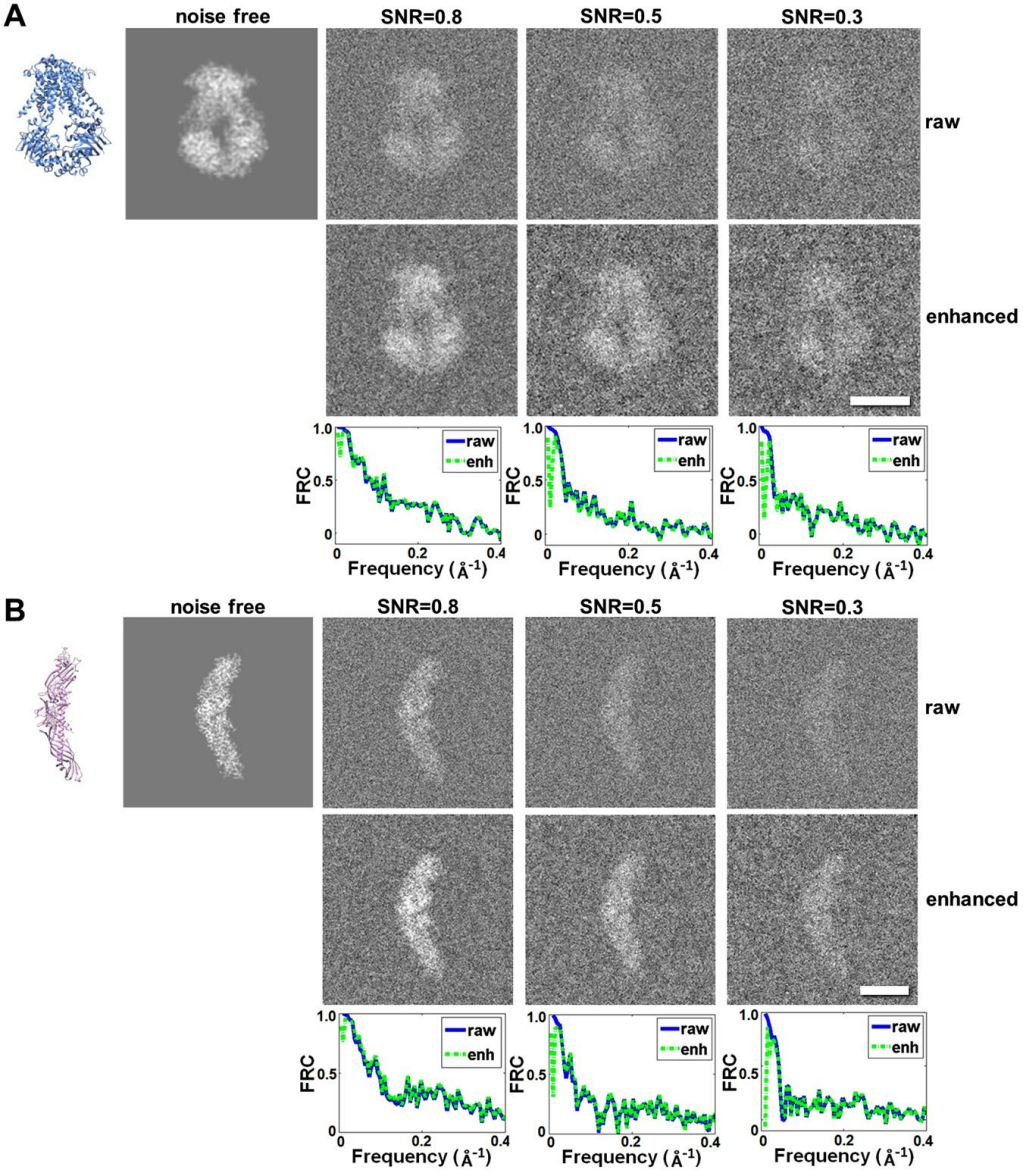
The effect of contrast enhancement on 2D images compared with raw data at different SNRs. (**A**) The projection images of ModB_2_C_2_ at SNR = 0.80, 0.50, and 0.30. The first row shows the raw images. The second row shows images after contrast enhancement. The third row shows the FRC curves between the corresponding raw images and their enhanced images. (**B**) The projection images and their corresponding enhanced images of CETP at SNR = 0.80, 0.50, and 0.30. Scale bars are 5 nm.

For ModB_2_C_2_, the Fourier ring correlation (FRC) curves calculated between the enhanced image and noise-free original reference image were similar to the FRC curves calculated between the non-enhanced images and noise-free original reference image (Fig. 2A). This result suggests that, although the enhancement method did not significantly change the image resolution, the image contrast was still significantly enhanced. The SNR levels increased by 60–70% compared with non-enhanced images, from 0.80 to 1.34, from 0.50 to 0.80 and from 0.30 to 0.53 (Table 1). Similar results were obtained for the small protein, CETP. The analyses showed that the FRC curves were also similar to the curves before applied the enhancement (Fig. 2B). The SNR levels were also increased by 60–70%, from 0.80 to 1.33, from 0.50 to 0.83 and from 0.30 to 0.49 (Table 2).

**Table 1:** SNR analyses of 2D images and 3D reconstructions of ModB_2_C_2_ before and after contrast enhancement.

Enhancement on the noisy images of simulated ModB_2_C_2_				Combination enhancement on the super high noisy images of simulated ModB_2_C_2_				
2D image		3D reconstruction		2D image			3D reconstruction	
raw	enhanced	raw	enhanced	raw	lp8Å	lp8Å + enh.	lp8Å	lp8Å + enh.
0.80	1.34	0.70	1.09	0.25	1.49	2.27	4.06	4.64
0.50	0.80	0.42	0.66	0.20	0.90	1.31	3.27	3.89
0.30	0.53	0.27	0.43	0.15	0.81	1.14	2.54	3.09

The SNRs of the simulation of ModB_2_C_2_. “2D” represents the SNR in untilted image, “3D” represents the SNR in the 3D reconstruction, “raw” stands for the initial image, and “enahnced” is for the enhanced image. “lp8Å” is for the image after low-pass filtered at 8 Å, and “lp8Å + enh.” is for the image after low-pass filtered at 8 Å and then enhanced.

**Table 2:** SNR analyses of 2D images and 3D reconstructions of CETP before and after contrast enhancement.

Enhancement on the noisy images of simulated CETP images				Combination enhancement on the super high noisy images of simulated CETP				
2D image		3D reconstruction		2D image			3D reconstruction	
raw	enhanced	raw	enhanced	raw	lp8Å	lp8Å + enh	lp8Å	lp8Å + enh
0.80	1.33	0.61	0.94	0.25	1.57	2.28	3.96	4.89
0.50	0.83	0.38	0.61	0.20	1.25	1.76	3.15	3.88
0.30	0.49	0.24	0.37	0.15	0.93	1.26	2.39	2.90

The SNRs of the simulation of CETP. “2D” represents the untilt images, “3D” represents the 3D reconstruction, “raw” is for the initial image, and “enhanced” is for the image after contrast enhancement. “lp8Å” is for the images after low-pass filtered at 8 Å, and “lp8Å + enh.” is for the images after low-pass filtered at 8 Å and enhancement.

The enhancement algorithm was also tested on experimental cryo-EM images of Termoplasma acidophilum 20S proteasome^31^ (Supplemental Fig. S1A, downloaded from Electron Microscopy Public Image Archive, entry EMPIAR-10025). These cryo-EM images were acquired on FEI Titan Krios TEM equipped with a Gatan K2 Summit direct detector, which has been used to achieve a 3D single-particle reconstruction at 2.8 Å resolution^31^. In the raw images, proteasome particles were barely visible (Supplemental Fig. S1A). After low-pass filtering the images at 8 Å, the particles became clear (Supplemental Fig. S1B), allowing us to easily box the particles from the images. One representative boxed particle showed a boost of SNR from ~0.06 to ~0.55 by the low-pass filtering (Supplemental Fig. S1C,D). The SNR of low-pass filtered images could be further increased to ~0.72 (~30% increase) by the enhancement algorithm (Supplemental Fig. S1E). The FRC curve between the raw image and the image after enhancement (also with low-pass filtered at 8 Å) is higher than 0.9 at frequency higher than 1/4 Å^−1^ (Supplemental Fig. S1F), demonstrating that the resolution of image was maintained by the enhancement algorithm. This result also evidenced that, although the density of image after enhancement does not reflect electron density of protein any more (due to the nonlinear boosting function of the enhancement), the information about protein structure was reserved in the enhanced images.

The above tests using both simulated and experimental 2D images showed that the enhancement procedure had no significant effect on the resolution of the images but increased the image contrast. The effect of contrast enhancement would not be reduced as the particle size decreased. Thus, the enhancement method can be used to improve the image constrast of 2D images of particles in various sizes.

### Implementation on the simulated 3D reconstruction

#### Testing the effect of the enhancement method on the simulated 3D reconstruction

It is well known that the 3D reconstruction resolution was significantly depended on two things, the noise level (image contrast) of the 2D tilt series and the accuracy of the alignment of those 2D tilt series. The noise can directly reduce the 3D reconstruction resolution and indirectly influence the accuracy of the alignment to further reduce the 3D reconstruction resolution. To improve the 3D reconstruction via increasing the accuracy of the alignment of the 2D tilt series, we conducted following processes on simulated data.

**Figure 3:**
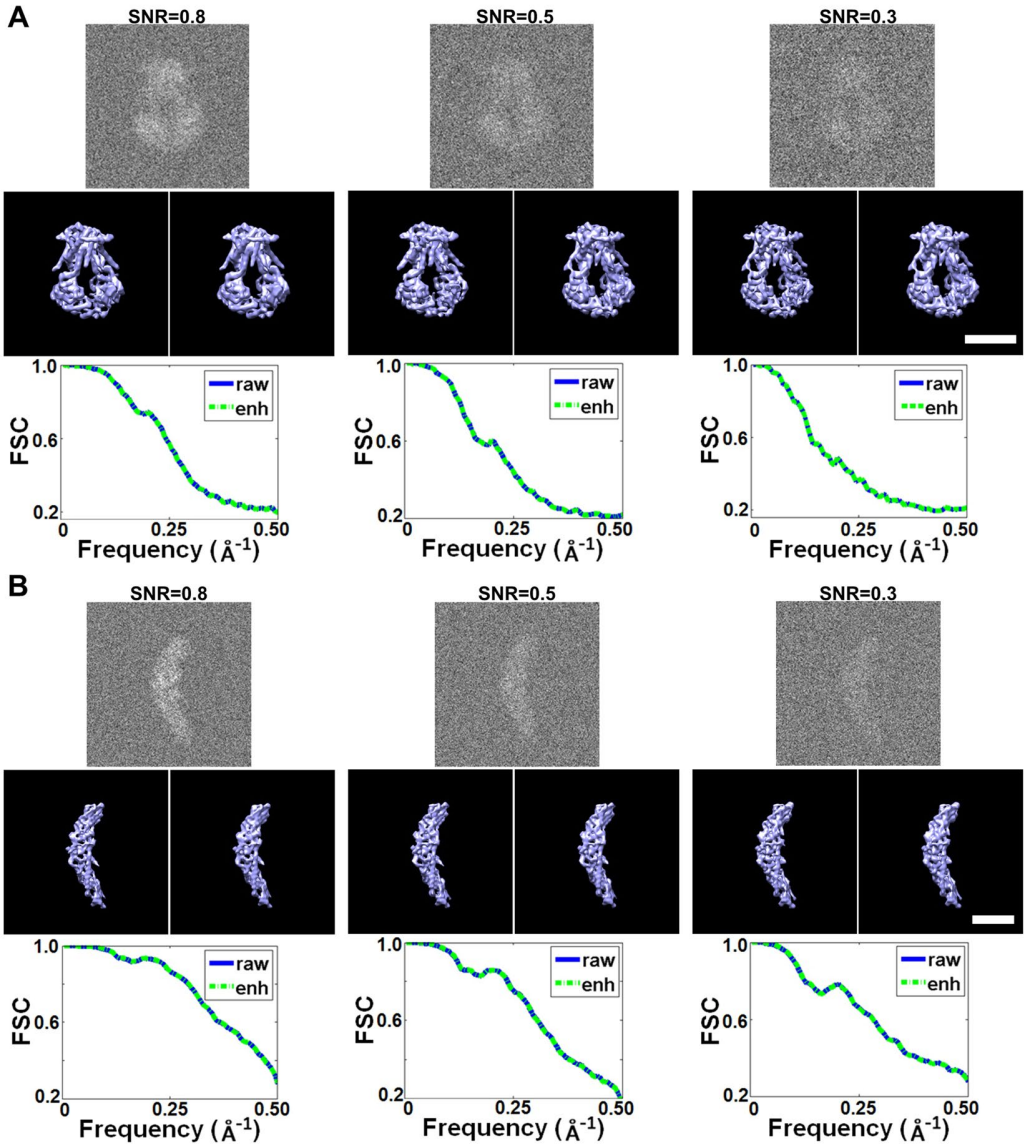
Comparison between reconstructions of raw and enhanced data. (**A**) The 3D reconstruction of ModB_2_C_2_ by back projection from tilt images set at SNR = 0.80, 0.50, and 0.30. The first row is the representative image of projections (at 0^°^ tilt). The second row is the 3D reconstructions from raw data (left) and enhanced data (right) (both maps were low-pass filterred to ~8 Å). The third row is the FSC curves of the 3D reconstructions using raw and enhanced data. (**B**) The 3D reconstruction of CETP at SNR = 0.80, 0.50, and 0.30 respectively. Scale bars are 5 nm.

A set of simulated tilt series (perfectly aligned, without image shift) was generated by projecting ModB_2_C_2_ from a tilt angle range of −90° to +90° in steps of 1° by using the “*PJ 3Q*” command (SPIDER software package)^30^. Gaussian noise was then added to the tilt series in SNRs of 0.80, 0.50, and 0.30 respectively. After contrast enhancement, each tilt series were directly back projected into a 3D reconstruction by using the “*BP 3F*” command (SPIDER software package). The Fourier shell correlation (FSC) curves computed between the 3D reconstruction and reference 3D object (noise free) showed that the enhancement method did not significantly change the 3D resolution nor change the structure (Fig. 3A); however, it did increase the SNR of the 3D reconstruction by approximately 50% (from SNR of 0.70 to 1.09, 0.42 to 0.66 and 0.27 to 0.43 respectively) (Table 1).

Similarly, by using a small protein, ~53 kDa CETP, 3D reconstructions were evaluated under the tilt series SNR levels of 0.80, 0.50, and 0.30 respectively. The FSC analyses of the 3D reconstructions showed no significant change in resolution (Fig. 3B), but the SNR levels were increased by 50%, (from 0.61 to 0.94, 0.38 to 0.61, and 0.24 to 0.37) (Table 2), which was consistent to the larger protein (ModB_2_C_2_).

The above evaluations showed that the enhancement method did not decrease the 3D reconstruction resolution and the reconstructed 3D map has similar features to that without enhancement, except few tiny differences (Fig. 3). However, the enhancement method improved the contrast of 3D reconstruction by ~50%. A further improvement in 3D constrast could be obtained by prior applied the low-pass filter before the enhancement. To demonstrate this approach, the images of ~108 kDa ModB_2_C_2_ samples were generated with super low SNRs, 0.25, 0.20 and 0.15, respectively. Each image was filtered by the low-pass filter at ~8 Å and then submitted to the enhancement. Statistical analyses showed that the contrast of 2D images were significantly improved after low-pass filterring as expected, *i.e*., the SNRs of the 2D images were increased from 1.49, 0.9 and 0.81 to 2.27, 1.31 and 1.14 respectively (Table 1). More importantly, the 3D reconstructions showed that the enhancement method led to less than 2% decreasing in the resolution (from ~7.9 Å, ~8.8 Å and ~10.8 Å to ~8.0 Å, ~8.9 Å and ~10.9 Å, respectively), but increased the 3D SNRs from 4.06, 3.27 and 2.54 to 4.64, 3.89 and 3.09 respectively (Table 1). The similar results were also obtained on small protein, CETP (Table 2). The SNRs of the CETP images at 0.25, 0.2 and 0.15 were increased to 1.57, 1.25, and 0.93 by low-pass filtering at ~8 Å, and then further increased to 2.28, 1.76, and 1.26 by the enhancement method. SNRs of the 3D reconstructions of CETP were increased from 3.96, 3.15, and 2.39 by low-pass filterring, and then further increased to 4.89, 3.88 and 2.90 respectively. The 3D resolutions were changed within a range of ~5%, *i.e*., from ~4.42 Å, ~5.75 Å, and ~7.73 Å to ~4.64 Å, ~5.95 Å and ~7.79 Å respectively. Therefore, the combination of low-pass filter and enhancement can benefit the 3D reconstructions of super high noisy images, such as the low-dose cryo-ET images.

#### Evaluating the enhancement on IPET 3D reconstruction of simulated electron tomography

**Figure 4:**
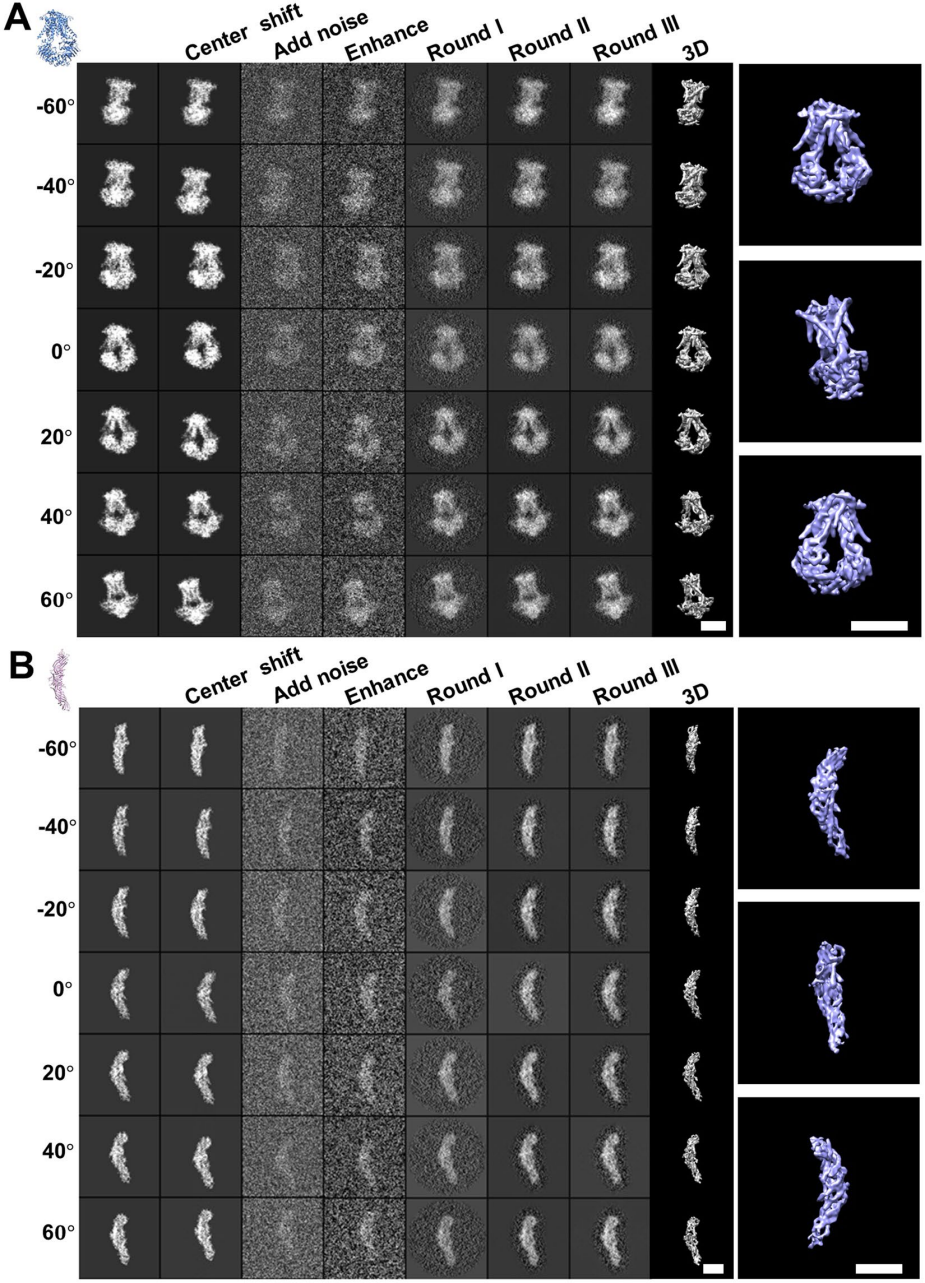
Simulating cryo-ET images and the corresponding IPET 3D reconstruction under the contrast enhancement condition. (**A**) The step-by-step process of the refinement and the final 3D reconstruction of a simulated particle of ModB_2_C_2_. The initial tilt images are projected from the model, and then random shifted and added with the noise (SNR = 0.30). After contrast enhancement, the images were reconstructed by using the IPET method. Seven representative tilted views are shown in the columns. The 3D reconstruction was displayed after low-pass filtering at 8 Å. The right panels are the tilted views of the final reconstruction. (**B**) Refinement procedures and IPET 3D reconstruction of a simulated particle of CETP. Scale bars are 5 nm.

The resolution of 3D reconstruction was depended on both the contrast of 2D tilt images and the accuracy of the image alignment. To evaluate the accuracy of the alignment of the enhanced images in IPET 3D reconstruction of an individual particle of protein (no averaging on different particles of same type of protein), we applied the method to a set of simulated tilt series of ET images of a single molecule, a ModB_2_C_2_ molecule. In this tilt series, the center of each tilt image was randomly shifted away from the center within a range of 30 pixels to simulate the translational errors. The enhancement process was applied to the simulated tilt series with SNR = 0.3 before IPET 3D reconstruction^1^. The process of IPET 3D reconstruction (Fig. 4A) showed that, as the alignment of the enhanced images was gradually improved, the contrast of the 3D reconstruction was gradually enhanced. The final 3D reconstruction showed the features of the secondary structure, such as the α-helix.

To further test the effect of the enhancement method on the IPET 3D reconstruction of small protein, CETP, the tilt series with SNR = 0.30 and center-shifted range of 30 pixels was generated, then submitted for enhancement before conducting the IPET 3D reconstruction (Fig. 4B). The process showed that the tilt images were systematically enhanced and aligned. The final 3D reconstruction confirmed that the high-resolution details could be reconstructed after the enhancement (Fig. 4B).

The above evaluations showed that images after enhancement could be well aligned for achieving a 3D reconstruction without losing the high resolution structure details, even for a small protein like CETP under a high noise condition. The results suggested that the enhancement method did not bias the image alignment thus can be used to improve the quality (SNR) of IPET 3D reconstruction of a single molecule.

### Implementation on experimental 3D reconstruction

#### Evaluating the enhancement on the IPET 3D reconstruction of negative-staining electron tomography

After confirmed the enhancement method regarding that it did not decrease the resolution of 3D reconstruction but increase the SNR on simulation data, we applied the method to the real experimental data. In this section, we applied the method to a set of high contrast negative staining tilt series of an 84-base-pair double-stranded DNA (dsDNA, with a molecular mass of ~52 kDa) conjugated to 5 nm nanogold. In the next section, we applied the method to a set of low contrast tilt series, *i.e*., a set of cryo-EM tilt series of a particle of LDL bound to CETP^32^ (the molecular mass of LDL and CETP are ~2,500 kDa^33^ and ~53 kDa, respectively), and to a set of cryo-PS tilt series of a particle of DNA origami acquired by using direct detector.

**Figure 5:**
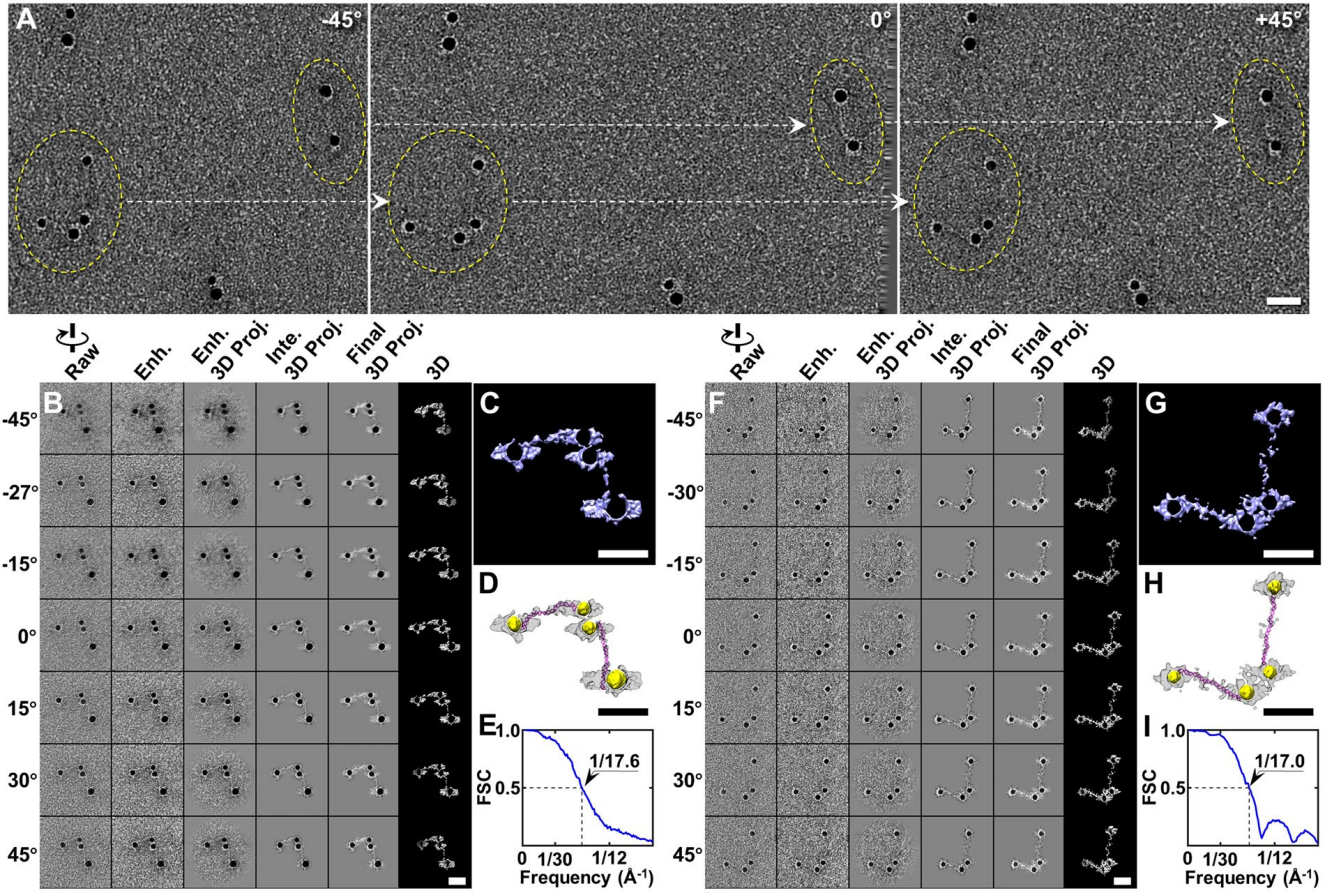
3D reconstruction of two representative double DNA-nanogold conjugates by IPET. (**A**) Three representative views of OpNS DNA-nanogold conjugates samples imaged using ET from a single-axis tilt series (from −60° to +60° at 1.5° intervals). Two DNA-nanogold particles (circled in yellow) with their orthogonal views are indicated by the dashed arrows in the three selected ET tilt micrographs (band-pass filter between 10 Å and 1500 Å). (**B**) Seven representative tilt images and enhanced images of one individual DNA-nanogold conjugates are displayed in the first two columns from the left. Using IPET, the tilt images (after CTF correction) were gradually aligned to a common center for 3D reconstruction by an iterative refinement process. The projections of the intermediate and final 3D reconstructions at the corresponding tilt angles are displayed in the next two columns according to their corresponding tilt angles. (**C**) Final IPET 3D density map of the targeted individual particle (after low-pass filtering at 16 Å). (**D**) The final 3D density map and its overlay with flexibly docked 84-bp dsDNA (final map in gray and its reversed map in gold). (**E**) The FSC curve between two density maps reconstructed from odd and even numbers of tilt images shows that the resolution of the IPET 3D density map was ~17.6 Å. (**F**–**I**). The 3D density map of a second individual DNA-nanogold conjugates was reconstructed from the tilt images using IPET. The FSC analysis showed that the 3D reconstruction resolution was ~17.0 Å. Scale bars are 20 nm.

The high contrast tilt series of a dsDNA-nanogold conjugate was imaged from −60° to +60° in steps of 1.5° ^3^. The three representative survey views showed each fiber-shaped dsDNA bound to two nanogold particles from each of its distal ends (Fig. 5A). Before the targeted particle was tracked and boxed from the tilt series, the CTF of the tilt series was measured and corrected by TomoCTF software package^34^. The single complex of dsDNA-nanogold conjugates was previously published; thus, the double complexes of the dsDNA-nanogold conjugates were used here for 3D reconstruction. The selected tilt view of the double complexes showed that the dsDNA portions were scarcely visible (left column in Fig. 5B) and the SNRs were within the range of ~0.17 to ~0.70 with an average of ~0.46. By the enhancement process, the SNRs was slightly increased to the range from ~0.22 to ~0.85 with an average of ~0.56, and the overall shape of the dsDNA became slightly visible (Fig. 5B, left second column). Through the IPET 3D reconstruction, the final 3D image showed two handcuff-shaped complexes attached to each other (Fig. 5C). The FSC analysis showed the 3D map had a resolution of ~17.6 Å (Fig. 5E). The overall conformation of each complex was similar to that previously published^3^. However, two complexes seemed attached to each other, owing to the interaction of irregularly shaped densities coating the surfaces of the nanogold particles (Fig. 5C). These densities may possibly have been thiolated short-chain polyethylene glycol (PEG) molecules that were used to stabilize the particles against aggregation at high ionic strength^35^. To reveal the approximate conformation of the dsDNAs, the standard model of 84-base-pair dsDNA was manually and flexibly docked into the fiber-shaped density between two nanogold particles in each complex (Fig. 5D). Because of the opposite image contrast of the nanogold particles relative to that of the DNA, we reversed the image contrast of the final 3D image (colored in gold) and overlaid this 3D image over the original 3D image to display both the DNA and nanogold particles in the same 3D map (Fig. 5D). This overlaid map showed nanogold particles with diameters of ~79.0 Å, ~59.0 Å, ~56.0 Å and ~65.0 Å. Each of the two high-density fabrics with overall dimensions of ~240.0 Å long and ~15 to ~25 Å wide bridged two nanogolds (Fig. 5D).

Similarly, another 3D density map of double complexes of dsDNA-nanogold conjugates was reconstructed by combining the enhancement method and IPET (Fig. 5F–I). The enhancement method slightly increased the SNRs of each tilt image from ~0.10 to ~0.47 (with an average of 0.27) to ~0.14 to ~0.54 (with an average of ~0.31). The 3D reconstruction showed that two handcuff-shaped particles were connected to each other by the surface interaction of two nanogolds in each complex. The ~17 Å resolution was measured on the basis of FSC analyses (Fig. 5I); the conformation was similar to that of the first double complexes. The overlaid density map from the final 3D density map and its reversed density map (colored in gold) showed that the nanogold particles had diameters of ~54.0 Å, ~63.0 Å, ~58.0 Å and ~60.0 Å, and were bridged by two fabric-like DNA densities with overall dimensions of ~255 to ~275 Å long and ~15 to 25 Å wide (Fig. 5H). The conformations of dsDNA strands were obtained by manually and flexibly docking the standard structure of 84-base-pair dsDNA models into the bridging portion density (Fig. 5H). Successfully repeating the previous IPET 3D reconstruction and achieving 3D reconstructions of the double complexes from negative-staining ET data confirmed that the enhancement method is a reliable method to preprocess tilt images before IPET 3D reconstruction.

The results obtained from the above process demonstrated a successful case in which the enhancement method benefits the IPET 3D reconstruction of a single target object based on the low contrast negative-staining images.

#### Evaluating the enhancement on the IPET 3D reconstruction of cryo-electron tomography

**Figure 6:**
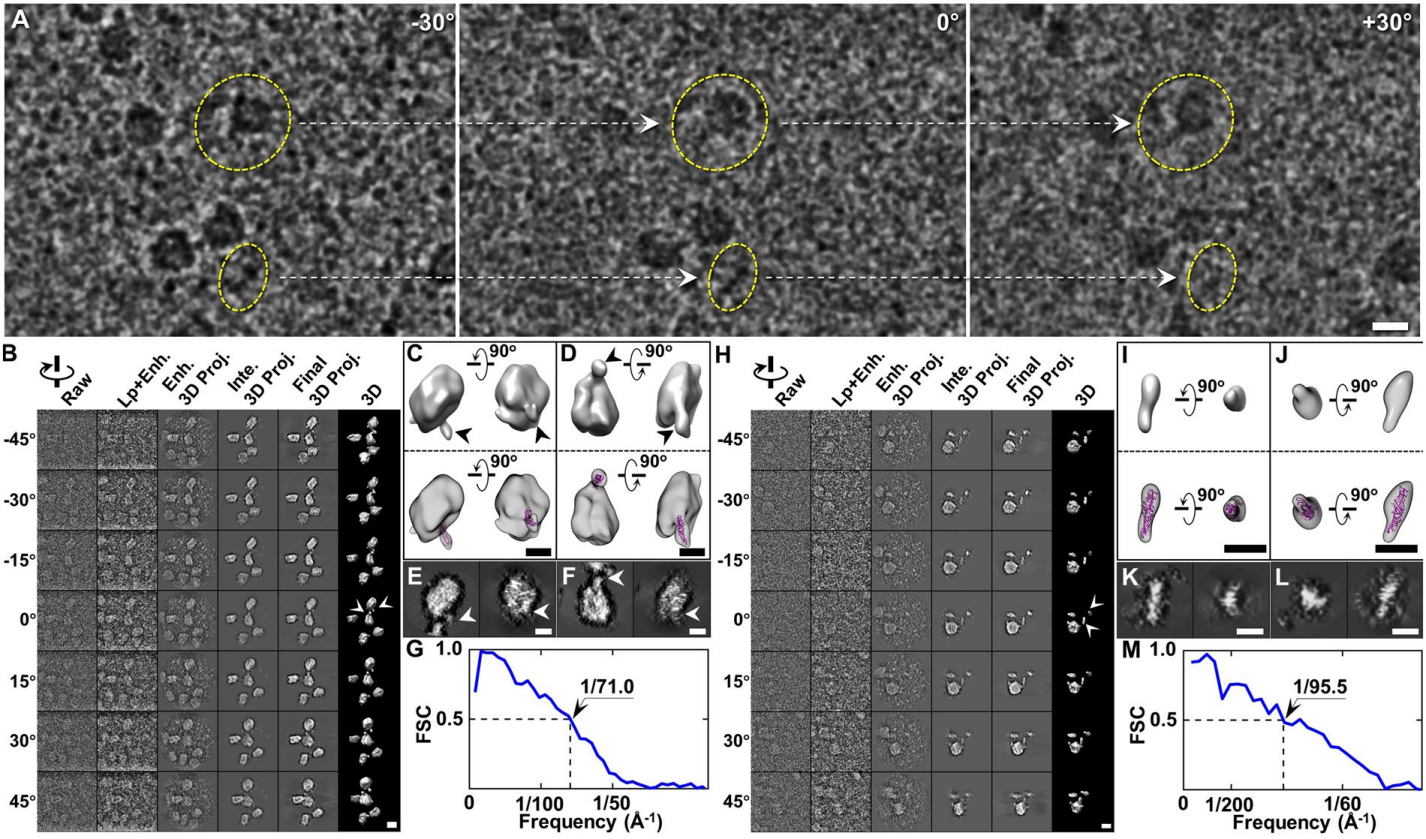
3D tomogram of the LDL-CETP complex by cryo-ET. (**A**) Three representative views of the single-axis tilt series of frozen hydrated LDL and CETP mixtures (band-pass filter between 50 Å and 1500 Å). (**B**–**G**) Refinement procedures and results from the first LDL-CETP complex (image contrast reversed). (**B**) IPET refinement procedures. (**C**,**E**) Two orthogonal views of one refined particle low-pass filtered at 70 Å shown as an iso-surface representation (**C**, top), with a docked CETP model (**C**, bottom) and as a re-projection (**E**). (**D**,**F**) Two orthogonal views of another refined particle low-pass filtered at 70 Å shown as an iso-surface representation (**D**, top), with a docked CETP model (**D**, bottom), and a re-projection (**F**). (**G**) The resolution was estimated on the basis of the FSC curve between two models built from odd- and even-numbered views. (**H**–**M**) IPET 3D reconstruction procedures of a second LDL-CETP complex. Scale bars are 20 nm in A, B, and H, and 10 nm in C to F, and I to L.

To evaluate the effect of the enhancement method on real experimental data with super low image contrast, we applied the method to a set of tilt series of cryo-EM images of a particle of LDL bound to CETP^32^. The tilt series of cryo-ET images was acquired from a series of tilting angles from −57° to +57° at 1.5° increments, under a low-dose condition (a total dose of ~25 e^−^/Å^2^) and a magnification of 50 k × (corresponding to 2.4 Å/pixel). The survey of cryo-ET micrographs at 3 representative angles showed the LDL and CETP particles from orthogonal views (Fig. 6A). Remarkably, the CETP’s molecular weight was significantly below the limitations of cryo-EM; the rod-shaped CETP could scarcely be seen on the surface of LDL, and in the background, vitreous ice, such as a LDL-CETP complex and a CETP alone particle, were masked for visualization. To confirm that the rod-shaped particles were the protein signal instead of the noise of the image, the entire tomography (after CTF correction by the TomoCTF software package^34^) was aligned and reconstructed with IMOD software^36^. Although the IMOD 3D density maps were too noisy to distinguish the LDL and CETP particles from the background, the projection along the Z direction of the 3D reconstruction provided sufficient contrast to evidence that the LDL alone, LDL-CETP complex, and even CETP alone were visible (Supplemental Fig. S2).

In IPET 3D reconstruction, the extremely low image contrasts (only ~0.16 to ~0.40 with an average of ~0.29) result in extremely challenging alignments. By combining a low pass filtering at 8 Å and the enhancement method, we increased the SNRs to a range of ~0.27 to ~0.65, with an average of ~0.47. These image contrasts provided a sufficient signal for IPET 3D reconstruction. The tilt images were gradually and iteratively aligned to their global center (Fig. 6B), and 3D density maps (after low-pass filtering at 70 Å) were reconstructed. The FSC analysis showed that the 3D resolution was ~71.0 Å based on the FSC 0.5 criterion (Fig. 6G). The 3D maps of two represented particles in the reconstructions were displayed from two perpendicular viewing directions, wherein an ellipsoidal particle (~260.0 Å × ~220.0 Å × ~150.0 Å) with flat opposing surfaces was attached to protrusions with length of ~110.0 Å and ~85.0 Å (Fig. 6C,D). The protrusions are slightly shorter than the crystal structure of CETP, suggesting that the portion of one distal end of CETP penetrated the surface of LDL, which is consistent with previous observations from the negative-staining method^37^. This test indicated that a single molecule of 53 kDa CETP imaged by cryo-ET could be reconstructed by using our approach.

To further evaluate whether an individual CETP could be reconstructed, we repeated the above process on another local area of the tilt series, where the images contained some free rod-shaped particles. The SNRs of the tilt series of images were only ~0.14 to ~0.28 with an average of ~0.21. After low pass filtering and enhancement, SNRs were increased to ~0.25 to ~0.47 with an average of ~0.36. Through the IPET reconstruction process, the tilt images were gradually and iteratively aligned to their global center (Fig. 6H). The 3D resolution was ~95.5 Å, on the basis of the 0.5 FSC criterions (Fig. 6M). The 3D reconstruction contained a large particle with rod-shaped protrusions, and some free rod-shaped particles (Fig. 6H). The large particle was ~260.0 Å × ~260.0 Å × ~150.0 Å, and the protrusion was ~85.0 Å in length (last column in Fig. 6H). Remarkably, two rod-shaped particles were also reconstructed (top row in Fig. 6I,J), which were ~140 to ~160 Å long that similar or slightly longer than the CETP crystal structure. The CETP molecules fit well when docking into the rod shapes as the best fit in the density map (bottom row in Fig. 6I,J). The shape and surface of CETP were also visualized by the corresponding projections (Fig. 6K,L). The similar shapes and dimensions of the rod-shaped particles to that of the crystal structure of CETP suggest that our enhancement method can achieve 3D reconstruction of a single molecule of 53 kDa CETP, although the CETP molecule has a molecular weight nearly one-third the minimum limit for cryo-EM.

**Figure 7:**
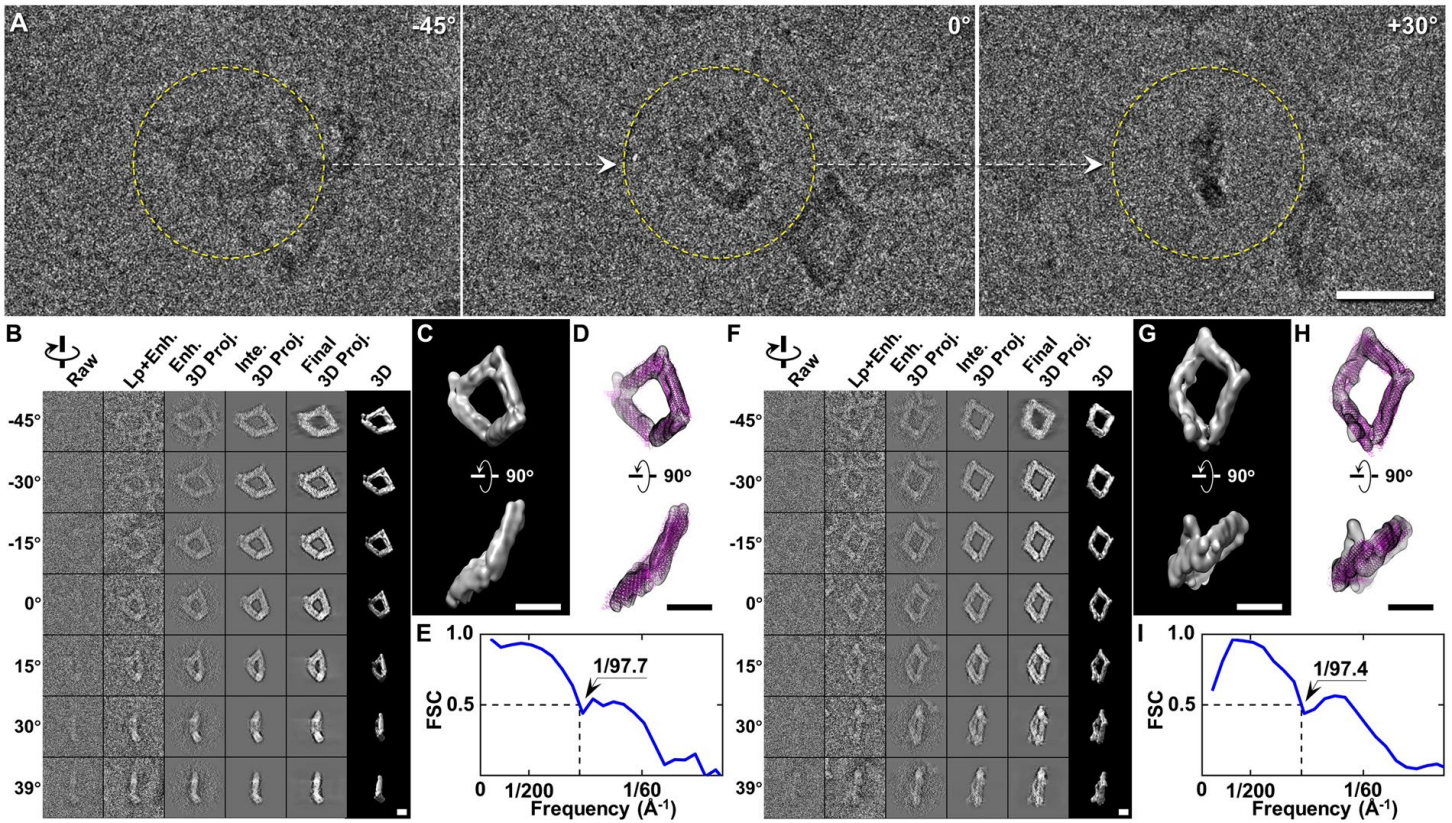
3D tomogram of the DNA origami by cryo-PS-ET. (**A**) Three representative views of the single-axis tilt series of frozen DNA origami (band-pass filter between 10 Å and 1500 Å). (**B**) IPET reconstruction procedures of a targeted particle of DNA origami (image contrast reversed). (**C**) Two orthogonal views of the IPET 3D map after low-pass filtered at 80 Å. (**D**) A DNA origami model was flexibly docked into the 3D map. (**E**) FSC curve shows the resolution of IPET reconstruction is 97.7 Å. (**F**–**I**) IPET 3D reconstruction procedures and result for a second particle of DNA origami. Scale bars are 50 nm in A, and 20 nm in B to D, and F to H.

The enhancement method was finally examined on a tilt series of DNA origami cryo-PS sample imaged by using Gatan K2 Summit direct electron detector. This tilt series was acquired from −48° to +48° at 1.5° increments under a magnification of 19 k × (corresponding to 1.85 Å/pixel). Three representative tilt images showed overall square-shaped DNA origamis (Fig. 7A). It should be pointed out that IPET reconstruction of these DNA origami particles was previously published^2^, in which only a low-pass filter was used to enhance image contrast. Here, to evaluate our proposed enhancement algorithm, the low-pass filtering was conducted before enhancement and IPET reconstruction. The contrasts of images were improved by both low-pass filtering and enhancement method, *i.e*., the average SNR of images increased from ~0.13 to ~0.23 by low-pass filtering, and then increased to ~0.36 by enhancement. Using those contrast enhanced images, a 3D map at a resolution of ~97.7 Å was obtained (Fig. 7B,E). The 3D map showed an overall ~70 nm quadrilateral shape particle (Fig. 7C) formed by four ~35-nm-long arms. By flexibly docking, an origami model could be obtained from a 3D map as a conformation of DNA origami (Fig. 7D). Using the same protocol, another 3D map at a resolution of 97.4 Å was also obtained from another targeted particle (Fig. 7F,I). The map allowed us to determine another conformation of DNA origami. These features of the density maps were very similar to the previous publication^2^, in which the low-pass filter was only used to increase the image contrast.

The edge-preserving image enhancing algorithm with the boost function was nonlinear function in changing the density of the image, which did not reflect the electron density of the protein. However, the FSC analysis on the 2D images and 3D reconstruction did not show the influence to the resolution or structure features on both simulation and experimental results. The possibilities for this phenomena could be the resolution obtained at current stage was not sufficiently high enough to show the influence, or the intensity obtained from EM did not truly reflect to the density of the protein due to multiple scattering of electron beam in electron microscopic imaging^38,39^, and/or element electron scattering was not linearly related to the mass of element due to elastic and inelastic scattering factors^40–42^. Nevertheless, the method increased the image contrast without reducing the 3D resolution at intermediate resolution showed the positive sign about the method.

## Conclusion

In this paper, an image contrast enhancement method was developed to increase the SNR before 3D reconstruction. The method was not a replacement method to current filters (such as low-pass filter or band-pass filter), but can be used as a supplementary tool to support the current filters to increase the SNR by ~20%, and hence allowed for successful 3D reconstruction of an individual particle of macromolecule. Through providing a substantial benefit for cryo-EM image reconstruction, this pre-process method may aid in the study of protein dynamics via 3D structures determined from each individual particle for revealing the structure variety among different targeted particles of the same type of protein.

## Methods

### Preparation of OpNS-EM, cryo-EM, and cryo-PS specimens

The NS specimens were prepared by the OpNS protocol^3^. An aliquot (∼4 μl) of the DNA-nanogold sample at a concentration of ∼20 μg ml^−1^ was placed on a thin carbon-coated 200-mesh copper grid (Cu-200CN, Pacific Grid-Tech, San Francisco, CA, USA) that had been glow-discharged. After ∼1 min of incubation, the excess solution on the grid was blotted with filter paper. Then, the grid was washed with water and stained with 1% (w/v) uranyl formate on Parafilm before being dried under nitrogen. The cryo-EM specimens were prepared as described previously^32^. LDL (produced by Children’s Hospital Oakland Research Institute) and CETP (produced by MERCK) were incubated at 37 °C for 15 minutes at a molar ratio of ~4:1. We used a high concentration of CETP to ensure the combination of LDL and CETP. An aliquot (∼3 μl) of the LDL-CETP mixture was placed on a glow-discharged holey-carbon grid (Cu-200HN, Pacific Grid-Tech, San Francisco, CA, USA). Then, the samples were flash-frozen in liquid ethane at ~90% humidity and 4 °C with a Leica EM GP rapid-plunging device (Leica, Buffalo Grove, IL, USA) after being blotted with filter paper. The cryo-PS specimens of DNA-origami were prepared as proposed by Zhang *et al*.^37^. An aliquot (~4 μL) of the DNA origami sample at a concentration of ~4 nM was placed on a glow-discharged lacey-carbon grid (LC200-Cu, Electron Microscopy Sciences, Hatfield, PA, USA) for ~1 min. The grid after washing by 1% (w/v) uranyl formate was then flash-frozen in liquid ethane at ~90% humidity and 4 °C with the Leica rapid-plunging device.

### ET data acquisition and image pre-processing

EM imaging of DNA-nanogold conjugates and LDL-CETP mixtures were conducted using a Zeiss Libra 120 transmission electron microscope (Carl Zeiss SMT GmbH, Oberkochen, Germany). The TEM was operated at 120 kV. A single-axis tilt series of the DNA-nanogold was collected from −60° to +60° in steps of 1.5° at a nominal magnification of 125 k × (0.94 Å/pixel). The LDL-CETP cryo-ET tilt series were collected from −57° to +57° in steps of 1.5° at a nominal magnification of 50 k × (2.4 Å/pixel). The tilt series were acquired at ~1 μm defocus using a 4 k × 4 k Gatan UltraScan 4000 CCD camera. For the cryo-ET, the electron dose per tilt series was within ~25 e^−^/Å^2^. Low-dose data acquisition was conducted by using the TEM tomography software (Gatan Inc., Pleasanton, CA, USA) in Advanced Tomography mode.

EM imaging of DNA origami was conducted using a FEI Tecnai TF20 TEM operated at 200 kV. A single-axis tilt series of the DNA origami was collected from −48° to +48° in steps of 1.5° at a nominal magnification of 19 k × (1.85 Å/pixel). The tilt series were acquired at ~1 μm defocus using a Gatan K2 Summit direct electron detector, and the total electron dose was ~50 e^−^/Å^2^.

### IPET 3D reconstruction

The defocus value of each tilt image was calculated by using the programs tomoctffind.exe in the TomoCTF software^34^. The phase of the tilt series was corrected by the program ctfcorrect.exe in the TomoCTF software. To reconstruct the 3D structures of individual particles, we first boxed the images of the particle from each tilt series of micrographs. The boxed images were then binned (DNA-nanogold conjugates for 3 times, LDL-CETP mixture and DNA origami for 2 times) to reduce computation time, and submitted to IPET 3D reconstruction^1^. The 3D reconstructions were finally submitted to a missing-wedge correction processing. The resolution was defined on the basis of FSC, when the frequency first decreased to a value of 0.5. The FSC curve was calculated by two 3D reconstructions generated from odd- or even-numbered index-aligned images. The SNR in both the 2D image and 3D map was calculated using the equation SNR = (I_*s*_ − I_*b*_)/N_*b*_, where I_*s*_ is the average intensity inside the particle, I_*b*_ is the average intensity outside the particle, and N_*b*_ is the s.d. of the noise calculated from the background (s.d. outside the particle).

### Program availability information

Software is available free of charge to academic end users.

## Electronic supplementary material

Support Information

Supplementary Table 1

## Data Availability

The datasets generated and/or analyses during the current study are available from the corresponding author on reasonable request. The IPET 3D density maps are available from the EM data bank (The NS density maps of DNANG: EMD-9262 and -9263; The cryo-EM density maps of LDL-CETP: EMD-9268 and -9269; The cryo-EM density maps of CETP alone: EMD-9270 and -9271; The cryo-PS density maps of DNA origami: EMD-9266 and -9267). Detailed experimental conditions is listed in Supplementary Table 1.
