# Supplementary material for: An Algorithm for Enhancing the Image Contrast of Electron Tomography
Source: Sci Rep. 2018 Nov 12;8:16711. doi: 10.1038/s41598-018-34652-9 (PMC6232092; doi:10.1038/s41598-018-34652-9)
Supplement: Supplementary file 1 — Support Information [file 41598_2018_34652_MOESM1_ESM.pdf]

Supplementary information

for

An Algorithm for Enhancing the Image Contrast of Electron Tomography

Hao Wu, Xiaobo Zhai, Dongsheng Lei, Jianfang Liu, Yadong Yu, Rongfang Bie, Gang Ren

Supplementary Figure

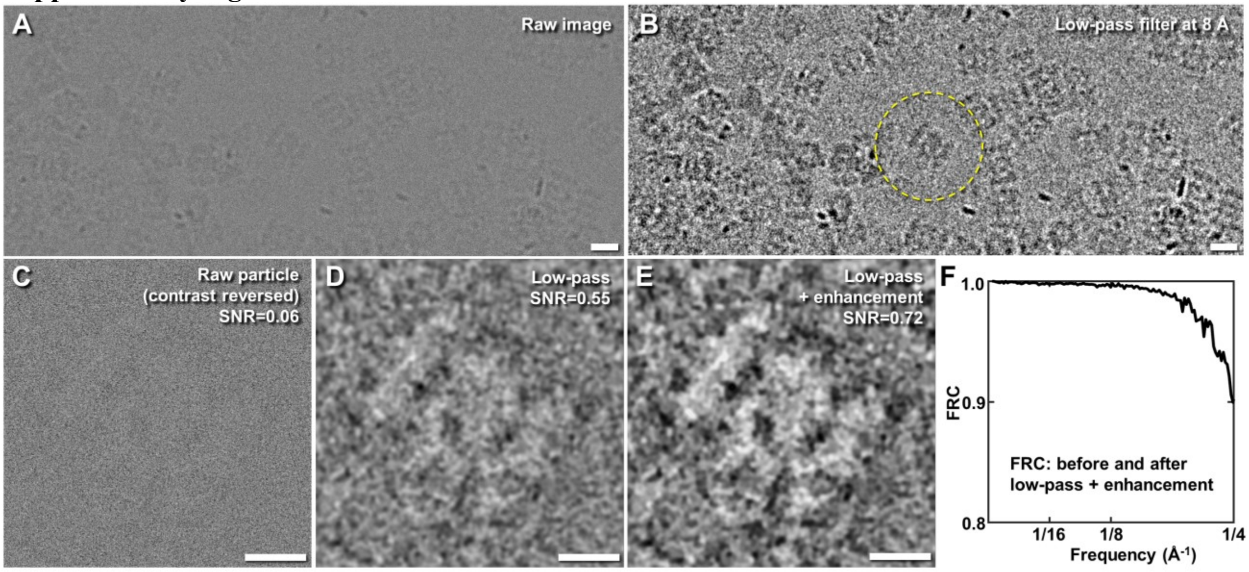

Supplementary Fig. S1 | The effect of contrast enhancement on 2D cryo-EM images. (A) Overview of one representative cryo-EM image of Termoplasma acidophilum 20S proteasome (downloaded from Electron Microscopy Public Image Archive, entry EMPIAR-10025). This image was acquired on FEI Titan Krios TEM equipped with a Gatan K2 Summit direct detector, and had been used to achieve a 3D single particle reconstruction at 2.8 Å resolution. (B) The image became clear after a low-pass filtering at 8 Å, allowing us to determine the position of particle easily. (C) A particle was boxed out from raw image (without low-pass filter) based on its position in low-pass filtered image. (D) The particle image was low-pass filtered at 8 Å. (E) The low-pass filtered particle image was submitted to enhancement. (F) The high FRC value between the raw image and image after enhancement suggests that the information of particle structure was well preserved by enhancement algorithm. Scale bars are 10 nm in A and B, and 5 nm in C to E.

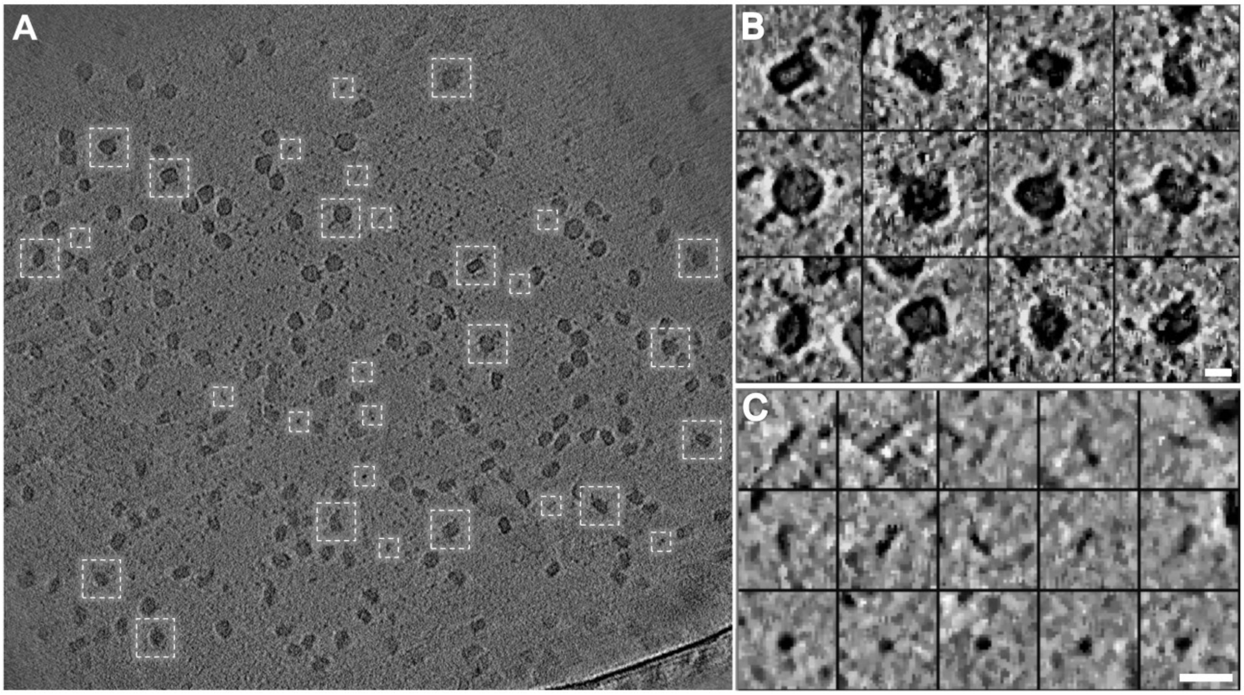

Supplementary Fig. S2 | The projection of 3D reconstruction of LDL-CETP by IMOD. (A) The tilt series of LDL-CETP sample was imaged from −57° to +57° in steps of 1.5° by cryo-ET. The large micrographs were aligned, and the 3D image was reconstructed by using IMOD software. The projection of the 3D image was conducted by using SPIDER software. (B) Representative LDL particles bound with CETP. For clarity, the images were processed by a median filter. (C) Representative CETP particles. Scale bars are 10 nm.
