# Supplementary material for: An Algorithm for Enhancing the Image Contrast of Electron Tomography
Source: Sci Rep. 2018 Nov 12;8:16711. doi: 10.1038/s41598-018-34652-9 (PMC6232092; doi:10.1038/s41598-018-34652-9)
Supplement: Supplementary file 2 — Supplementary Table 1 [file 41598_2018_34652_MOESM2_ESM.docx]

**Supplementary information**

**For**

**An Algorithm for Enhancing the Image Contrast of Electron Tomography**

Hao Wu, Xiaobo Zhai, Dongsheng Lei, Jianfang Liu, Yadong Yu, Rongfang Bie, Gang Ren

**Supplementary Table 1**

| **#** | **Sample** | **EMDB#^1^** | **TEM^2^** | **CCD^3^** | **Mag.** | **Apix^4^ (Å)** | **Dose/img.^5^**  **(e^-^/Å^2^)** | **Dose/set^6^**  **(e^-^/Å^2^)** | **Acq. angle range^7^** | **Total img.^8^** | **Reconst.**  **angle range^9^** | **Cont.^10^** | **Resol.^11^ (nm)** | **Fig. ^12^** |
| --- | --- | --- | --- | --- | --- | --- | --- | --- | --- | --- | --- | --- | --- | --- |
| 1 | DNA-NG #1 | EMD-9262 | Zeiss 120 | UltraScan | 125 kX | 2.82 | 54.64 | 4426.11 | -60˚ to +60˚ | 81 | -60˚ to +60˚ | 0.214 | 1.76 | Fig. 5C |
| 2 | DNA-NG #2 | EMD-9263 | Zeiss 120 | UltraScan | 125 kX | 2.82 | 56.39 | 4567.71 | -60˚ to +60˚ | 81 | -60˚ to +60˚ | 0.325 | 1.70 | Fig. 5G |
| 3 | LDL-CETP #1 | EMD-9268 | Zeiss 120 | UltraScan | 50 kX | 4.8 | 0.26 | 19.67 | -57˚ to +57˚ | 77 | -57˚ to +57˚ | 0.300 | 7.10 | Fig. 6C |
| 4 | LDL-CETP #2 | EMD-9269 | Zeiss 120 | UltraScan | 50 kX | 4.8 | 0.26 | 19.67 | -57˚ to +57˚ | 77 | -57˚ to +57˚ | 0.300 | 7.10 | Fig. 6D |
| 5 | CETP #1 | EMD-9270 | Zeiss 120 | UltraScan | 50 kX | 4.8 | 0.32 | 24.28 | -57˚ to +57˚ | 77 | -57˚ to +57˚ | 0.939 | 9.55 | Fig. 6I |
| 6 | CETP #2 | EMD-9271 | Zeiss 120 | UltraScan | 50 kX | 4.8 | 0.32 | 24.28 | -57˚ to +57˚ | 77 | -57˚ to +57˚ | 1.000 | 9.55 | Fig. 6J |
| 7 | DNA Origami #1 | EMD-9266 | FEI TF20 | K2 Summit | 19 kX | 3.7 | 0.69 | 44.64 | -48˚ to +48˚ | 65 | -48˚ to +40.5˚ | 0.296 | 9.77 | Fig. 7C |
| 8 | DNA Origami #1 | EMD-9267 | FEI TF20 | K2 Summit | 19 kX | 3.7 | 0.69 | 44.73 | -48˚ to +48˚ | 65 | -48˚ to +40.5˚ | 0.292 | 9.74 | Fig. 7G |
| **#** | **Sample** | **EMDB#^1^** | **TEM^2^** | **CCD^3^** | **Mag.^3^** | **Apix^4^ (Å)** | **Dose/img.^5^**  **(e-/Å^2^)** | **Dose/set^6^**  **(e-/Å^2^)** | **Acq. angle range^7^** | **Total img.^8^** | **Reconst.**  **angle range^9^** | **Cont.^10^** | **Resol.^11^ (nm)** | **Fig. ^12^** |

^1^ EMDB Index: <https://www.ebi.ac.uk/pdbe/emdb/>

^2^ TEM model: FEI TF20 stands for FEI TF200 TEM; Zeiss 120 stands for Zeiss Libra 120 Plus TEM

^3^ CCD: K_2_ Summit stands for Gatan K_2_ Summit Direct Detector; UltraScan stands for Gatan UltraScan 4000 4Kx4K CCD

^4^ Angstrom per pixel

^5^ Dose used for each CCD frame

^6^ Dose used for whole tilt series

^7^ Data acquisition angle range

^8^ Total images in the tilt series

^9^ Reconstruction angle range

^10^ Contour used for display

^11^ IPET 3D reconstruction resolution

^12^ The process of IPET 3D reconstruction showed in Figure
